# A Prospective Study of CPAP Therapy in Relation to Cardiovascular Outcome in a Cohort of Romanian Obstructive Sleep Apnea Patients

**DOI:** 10.3390/jpm11101001

**Published:** 2021-10-02

**Authors:** Ioana Maria Chetan, Anca Diana Maierean, Bianca Domokos Gergely, Georgiana Cabau, Raluca Tomoaia, Ana Florica Chis, Adriana Albu, Mirela Anca Stoia, Stefan Cristian Vesa, Dan Blendea, Doina Adina Todea

**Affiliations:** 1 Department of Pneumology, “Iuliu Hatieganu” University of Medicine and Pharmacy, 400332 Cluj-Napoca, Romania; mariaioana_25@yahoo.com (I.M.C.); biancadomokos@yahoo.com (B.D.G.); anna_f_rebrean@yahoo.com (A.F.C.); doina_adina@yahoo.com (D.A.T.); 2 Heart Institute “Nicolae Stancioiu“, 400001 Cluj-Napoca, Romania; raluca.tomoaia@gmail.com; 3 Department of Medical Genetics, “Iuliu Haţieganu” University of Medicine and Pharmacy, 400349 Cluj-Napoca, Romania; georgiana.cabau@gmail.com; 4 Department of Cardiology, “Iuliu Hatieganu” University of Medicine and Pharmacy, 400437 Cluj-Napoca, Romania; 5 2nd Internal Medicine Department, “Iuliu Hatieganu” University of Medicine and Pharmacy, 400000 Cluj-Napoca, Romania; adriana.albu@umfcluj.ro; 6 Department of Internal Medicine, “Iuliu Hatieganu” University of Medicine and Pharmacy, 400006 Cluj-Napoca, Romania; mirelastoia@yahoo.com; 7 Department of Pharmacology, Toxicology and Clinical Pharmacology, “Iuliu Hatieganu” University of Medicine and Pharmacy, 400337 Cluj-Napoca, Romania; stefanvesa@gmail.com; 8 Department of Medicine, Faculty of Medicine, “Iuliu Hatieganu” University of Medicine and Pharmacy, 400337 Cluj-Napoca, Romania; dblendea1@gmail.com

**Keywords:** cardiovascular disease, CPAP, obstructive sleep apnea syndrome, serum lipids, tricuspid annular plane systolic excursion

## Abstract

Background: Despite efforts at treatment, obstructive sleep apnea (OSA) remains a major health problem, especially with increasing evidence showing an association with cardiovascular morbidity and mortality. The treatment of choice for OSA patients is Continuous Positive Airway Pressure (CPAP), which has been proven in randomized controlled trials to be an effective therapy for this condition. The impact of CPAP on the cardiovascular pathology associated with OSA remains, however, unclear. Although the effect of CPAP has been previously studied in relation to cardiovascular outcome, follow-up of the treatment impact on cardiovascular risk factors at one year of therapy is lacking in a Romanian population. Thus, we aimed to evaluate the one-year effect of CPAP therapy on lipid profile, inflammatory state, blood pressure and cardiac function, assessed by echocardiography, on a cohort of Romanian OSA patients. Methods: We enrolled 163 participants and recorded their baseline demographic and clinical characteristics with a follow-up after 12 months. Inflammatory and cardiovascular risk factors were assessed at baseline and follow up. Results: Our results show that CPAP therapy leads to attenuation of cardiovascular risk factors including echocardiographic parameters, while having no effect on inflammatory markers. Conclusion: Treatment of OSA with CPAP proved to have beneficial effects on some of the cardiovascular risk factors while others remained unchanged, raising new questions for research into the treatment and management of OSA patients.

## 1. Introduction

Obstructive sleep apnea (OSA) is a common sleep disorder that affects about 4–7% of the general adult population, with reported global prevalence of 1 billion persons affected and an estimated prevalence exceeding 50% in some countries. In Romania, data regarding the clinical features of OSA, and mainly the burden of comorbidities, are lacking. According to a recent study, the estimated prevalence of OSA in Romania is 48.1% [1,2]. Presence of recurrent partial or complete collapse of the upper airway during sleep, leading to chronic intermittent hypoxia, is the main characteristic of the disease [3]. Among known risk factors for sleep apnea, some are also risk factors well established for cardiovascular disease (CVD). There is growing evidence demonstrating that OSA patients have an increased risk of cardiovascular morbidity and mortality [4]. OSA has been associated with different forms of cardiovascular disease including heart failure, hypertension, arrythmias, stroke and coronary artery disease [5]. Despite the fact that there is a strong connection between CVD and OSA, randomized clinical trials are yet to show that treating OSA improves cardiovascular outcomes [6]. There is very limited data from randomized trials, which have been limited in number and design [7].

Pathophysiologic mechanisms connecting OSA with CVD include oxidative stress, endothelial dysfunction, increased sympathetic nervous system activity, predilection for hypertension, dyslipidemia and metabolic dysregulation (insulin resistance) [8]. OSA treatment has been proved to diminish these processes, offering a feasible mechanism by which therapy could influence cardiovascular (CV) outcomes [9].

Efficient management of OSA includes a comprehensive assessment of each individual’s characteristics, as well as monitoring and follow-up. Continuous positive air pressure (CPAP) therapy is a first-line treatment for all patients diagnosed with OSA. It is cost effective and has been shown to both reduce apnea/hypopnea index (AHI) and improve quality of life [10]. Nevertheless, CPAP continues to be affected by adhesion problems. Results of an extensive analysis based on the literature suggest that, despite many interventions planned to improve adherence rates over the long term, trends show no clinical changes on its impact, with a high nonadherence of 30–40% [11]. Early retrospective trials supported the effectiveness of OSA therapy in enhancing CV outcomes. [12,13,14]. The SAVE (Sleep Apnea Cardiovascular Endpoints) trial was the largest randomized control trial (RCT) done to evaluate whether CPAP therapy could improve CV outcomes in subjects with established CVD [6]. The trial did not demonstrate any significant reduction in the primary end points (composite of CV death, myocardial infarction, stroke, hospitalization, heart failure (HF) or transient ischemic attack), although significant/notable improvements were obtained in health-related quality of life, snoring and daytime sleepiness.

While CPAP therapy seems to be successful in reducing OSA symptoms, the findings of randomized trials, so far, do not support its effectiveness in reducing the risk of CV events in OSA patients [15]. Thus, these limitations may serve as topics of interest for future investigations. Furthermore, the Romanian National Health Insurance System does not reimburse the costs related to the investigation and management of OSA, so many patients remain undiagnosed, and without treatment, due to the costs.

We hypothesized that CPAP therapy improves lipid profile levels, along with blood pressure and RV function echocardiographic parameters. Moreover, we assumed that by identifying the effects of CPAP therapy on cardiovascular risk factors, we may improve awareness in the Romanian population about the importance of early diagnosis and subsequentl treatment of OSA, despite the costs. CPAP is the only treatment, at the present time, with evidence that supports improvement in quality of life and reduction of the burden of associated comorbidities.

Given these considerations, the objectives of our study were to provide current and reliable data regarding the effect of CPAP therapy at a one-year follow-up on cardiovascular risk factors in the OSA adult population in Romania, to estimate their prevalence, awareness and improved control.

## 2. Materials and Methods

### 2.1. Study Population

Between January 2018 and December 2019, we examined and screened a cohort of consecutive adult patients for OSA in our Pulmonology Clinic of Cluj-Napoca. The patients were admitted to the hospital because of a history of daytime sleepiness, apnea or snoring. The study was conducted according to the guidelines of the Declaration of Helsinki and was approved by the ethics committee (number: 103/2018); all patients provided written informed consent.

Inclusion criteria for patients were age ≥ 18 years, and the presence of a minimum of three clinical symptoms of OSA. The symptoms were snoring, witnessed apneas, gasping/choking episodes, excessive sleepiness not explained by other factors, nocturia, morning headaches and decreased concentration and memory.

We excluded patients with unstable or decompensated cardiopulmonary disease, malignancy, recent surgery, physical or psychological incapacity, chronic intake of hypnotics or refusal to participate in the study.

We enrolled 205 patients in the study who met the inclusion criteria, of whom 17 declined participation in the study, 23 could not be reached and two had died from noncardiac or respiratory causes.

The patients included were divided into two groups based on necessity of CPAP therapy. The initiation of therapy was done according to Medicare guidelines as follows: all patients with an AHI greater than 15 were considered eligible for CPAP, regardless of symptomatology; for patients with an AHI of 5–14.9/h, CPAP was indicated only if the patient had one of the following: excessive daytime sleepiness, impaired neurocognitive function, mood disorders, insomnia, cardiovascular disease (e.g.,: hypertension, ischemic heart disease), or a history of stroke [16].

**Figure 1:**
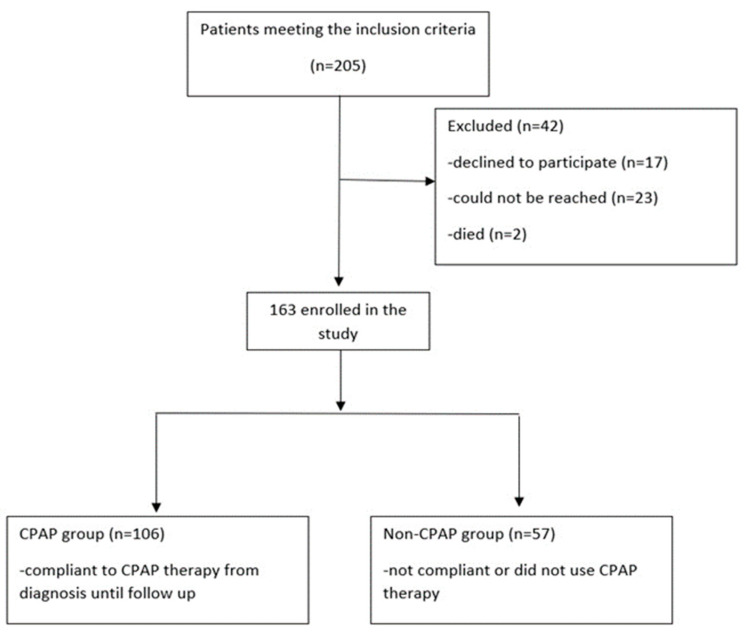
Study flow chart.

The first group included patients not using CPAP therapy, either because they did not match the specific criteria or because of noncompliance (non-CPAP group, n = 57). The second study group included patients who underwent CPAP therapy (CPAP group; n = 106) based on the results of a sleep study and symptomatology, as previously stated. Follow up data were collected from the 163 participants who completed the study (Figure 1).

Cardiovascular risk profile was assessed at the time of first visit. This included patient’s history, tobacco smoking history, medication use (lipid lowering drugs, antihypertensive agents), a physical exam, including anthropometric measurements, blood pressure, a sleep study, ECG, and echocardiography, and a blood exam (triglycerides, total cholesterol, C-reactive protein).

### 2.2. Sleep Study

All participants underwent a cardiorespiratory sleep study using a Nox T3 polygraphy device, which included continuous recording from nasal cannulae, heart rate, oxygen saturation, tracheal sounds (microphone), thoracic and abdominal movement and body position. Sleep study results were analyzed and approved by trained personnel. Apnea was defined as a complete cessation of airflow lasting at least 10 s, while hypopnea was diagnosed when there was reduction in respiratory airflow of >50% lasting 10 s or longer. The absence of airflow in the presence of paradoxical thoracic or abdominal motion was diagnosed as obstructive apnea [17]. The number of events of apnea and hypopnea per hour was calculated. OSA diagnosis was established in the event that AHI was greater than or equal to 15 per hour, or greater than or equal to 5 and less than or equal to 14 events per hour with documented symptoms of unintentional sleep episodes during wakefulness; daytime sleepiness; insomnia; mood disorders; the bed partner describing loud snoring, breathing interruptions, or both, during the patient’s sleep, or documented hypertension, ischemic heart disease or history of stroke, [16]. CPAP titration was made using an autoCPAP device (Philips respironics dreamstation AutoCPAP) after a validated protocol [18]. All participants received sleep hygiene advice and counseling for weight loss.

### 2.3. Echocardiography

Standard transthoracic echocardiography and Doppler evaluation were performed using commercially available equipment (iE33; Philips Medical Systems, Andover, MA, USA). All measurements were assessed, as an average of three consecutive beats according to the current European and American guidelines [19]. The following measurements were performed: basal right ventricle (RV) diameter in the apical four chamber view (A4C) at the end-diastole, tricuspid annular plane systolic excursion (TAPSE) as a parameter of RV longitudinal systolic function in a standard A4C; left ventricular ejection fraction (LVEF) and mean tricuspid regurgitant gradient. The left ventricle (LV) volumes (end-systolic volume [ESV] and end-diastolic volume [EDV]) and LVEF were measured using manual tracing (biplane Simpson’s) method.

### 2.4. Blood Pressure Assessment

Blood pressure (BP) was measured in the right arm using a standard mercury sphygmomanometer during each day of hospitalization, and an average of the values was obtained for further analysis. During all measurements, patients were supine and awake.

### 2.5. Anthropometric and Biochemical Measurements

Body weight and length were measured and recorded. Body mass index (BMI) was calculated with the formula of body weight/height^2^ (kg/m^2^). The same person performed all the measurements using the same tools. Obesity was defined as a body mass index (BMI) ≥ 30 kg/m^2^.

In all patients, fasting blood samples were drawn at the first visit and after 12 months, to evaluate serum levels of triglycerides (TG), total cholesterol (TC) and C-reactive protein (CRP). Serum samples were analyzed according to standard laboratory methods.

### 2.6. Outcome and Follow-Up

Patients had follow-up visits at 1, 6 and 12 months with clinical exams performed at each visit. Laboratory tests and a sleep study were performed at enrollment and at the 12 months follow-up visit. CPAP used at least 4 h per night for at least 70% of the days monitored was considered adequate adherence determined from CPAP tracking systems. Every medical visit considered appearance of any new medical events, changes in treatment, clinical variables and adherence to CPAP. Lipid lowering therapy and antihypertensive treatment were kept constant during the initial visit and follow up.

### 2.7. Statistical Analysis

Statistical analysis was performed using the MedCalc^®^ Statistical Software version 19.7 (MedCalc Software Ltd., Ostend, Belgium; https://www.medcalc.org (accessed on 12 January 2021)). Quantitative data were examined for normality of distribution using the Shapiro-Wilk test and were expressed as median and 25–75 percentiles. Qualitative data were expressed as frequency and percentage. Comparisons between groups were verified using the Mann-Whitney test or chi-square test, whenever appropriate. Comparison between baseline and follow-up, taking into account the treatment with CPAP, was performed with two-way ANOVA for the repeated measurements test after qualitative variables were log transformed. A “*p*” value lower than 0.05 was considered statistically significant.

## 3. Results

Median age of the study population was 59 (IQR 52.6; 65.2) with 45.6% identified as female in the non-CPAP group, and 61 (IQR 53.7; 67) with 35.8% female in the CPAP group. Among non-CPAP patients 38.6% were on treatment with lipid lowering drugs and 68.4% on antihypertensive treatment, comparative with CPAP group, where 34.9% were receiving lipid lowering drugs and 78.3% anti-hypertensive agents. Regarding smoking status, there was no significant difference at baseline between the groups, with 37 smokers in non-CPAP group (64.9%) and 57 smokers in the CPAP group (53.8%) (chi2 =1.872, *p* = 0.17). The median AHI was 44.3 (IQR 22.8; 82.1) in the non-CPAP group, and 45 (IQR 32.2;65.9) in group treated with CPAP. Median BMI for the study population at baseline was 38.1 (IQR 34.4; 40.7) in the non-CPAP group and 37.5 (IQR 33.08; 41.9) in the CPAP group, without significant differences between the two groups (*p* = 0.44). All baseline demographic and clinical characteristics of the study population are summarized in Table 1. Differences at baseline were observed only for SBP and DBP (*p* < 0.001; *p* = 0.01, respectively).

The follow-up studies were conducted 12 months following initiation of CPAP treatment. Respiratory polygraphy parameters at follow up were significantly improved in the CPAP group compared with the non-CPAP group. Median nadir oxygen saturation at one year was 90% in the CPAP group with AHI significantly reduced. The BMI decreased significantly from baseline to follow-up in the CPAP group (Table 2).

Following 12 months of CPAP therapy, TC levels were significantly decreased compared with the control group. Adjustment for age, BMI, sex, AHI, lipid lowering drugs and diabetes disclosed an independent influence of the CPAP therapy and TC serum levels (*p* = 0.001). In contrast, serum TG did not change significantly between the groups. Concerning the impact of CPAP treatment on the inflammatory state in the two groups, no changes were observed on the CRP levels at follow up.

When changes in blood pressure during the study period were compared between study groups (106 patients in the CPAP group, non-CPAP group, 57 patients), the CPAP group achieved a greater decrease in mean DBP at 12 months, but not in mean SBP levels. The model did not change when it was adjusted for potential confounding factors such as sex, age, antihypertensive treatment, BMI and AHI.

Comparing the OSA patients with and without treatment showed significant improvement of TAPSE. Regarding RV dimensions, there was no significant difference between the groups. Furthermore, the trans-tricuspid gradient was higher at follow up in the CPAP group compared with the non-CPAP group but slightly reduced when compared to baseline values. LV EF did not differ between the groups either at baseline or at the 12 months follow up.

## 4. Discussion

Despite recent interest in assessing the impact of CPAP therapy on cardiovascular risk factors, the research available so far includes retrospective, observational, and nonrandomized studies, and the results are unconclusive or conflicting. Our study is the first to explore the one-year cardiovascular effect of CPAP in a Romanian cohort of OSA patients.

In the current study, we found that treatment of OSA using CPAP therapy is independently associated with improvement in total cholesterol, diastolic blood pressure and TAPSE after adjusting for confounding factors such as sex, age, antihypertensive treatment, lipid lowering agents, BMI and AHI. On the other hand, TG, CRP, TAS, RA-RV gradient, RV dimensions and LVEF showed no considerable improvement.

### 4.1. Effect on Serum Lipids

The current results are partially confirmatory of previous research, such as the report by Xu et al. which-showed, in six RCTs (randomized controlled trials), that CPAP decreased TC levels in OSA patients, the decrease being dependent on age, BMI and duration of CPAP treatment, with younger, obese and longer duration patients benefiting the most, but no significant effect of CPAP on TG levels, which is in accordance with our results [20]. Another study demonstrated that application of CPAP therapy for as short as 2 months reduced TC and LDL-C levels in a cohort of patients with severe OSA, and the effect was sustained after 5 years of therapy [21]. On the other hand, Sharma et al. [22] described no significant improvement in lipid levels of OSA patients after 3 months of CPAP. Another study reported the same results but after 12 weeks [23].

A number of studies suggest that OSA is independently associated with lipid abnormalities [24,25,26], while others show that dyslipidemia is associated with obesity and not directly with OSA [27,28]. In an analysis of a selected group of patients with and without OSA, Kono et al. [29] showed that OSA was associated with dyslipidemia in nonobese patients. Changes in BMI during CPAP therapy may also influence lipid profile, and weight loss alone can determine a reduction in serum lipids [30]. In our study we demonstrated that the beneficial effect of CPAP therapy is independently associated with improvement of TC in OSA patients.

Despite the fact that the TC level improved after 12 months of therapy, there was no significant reduction in TG levels in the OSA group treated with CPAP therapy. These results are consistent with a number of investigations [20,21], but in opposition with the results of other studies [31,32]. The presence of dyslipidemia in OSA patients is based on several factors, including obesity, physical activity, OSA severity, nocturnal hypoxemia, sympathetic activity; therefore, sleep apnea is only one of the risk factors that can trigger dyslipidemia, which could explain the effect of CPAP therapy only on certain components of the lipid profile. Total cholesterol is one of the lipid components that is highly susceptible to the reduction of oxidative stress associated with continuous positive pressure treatment, as concluded by Robinson et al. [33]. At the same time, Imadojemu et al. showed that CPAP improves the sympathetic response to hypoxic chemoreflex stimulation, lowering serum the TC level [34], which could be another explanation for our findings.

### 4.2. CPAP and Markers of Inflamation

Previous studies showed that inflammatory processes may be involved in the pathway between OSA and dyslipidemia [35,36]. To explore the influence of inflammatory mediators we chose CRP, which plays important roles in inflammatory processes, being a well-established marker of inflammation, shown to be elevated in cardiovascular events [37]. We did not see a significant change in CRP levels after CPAP therapy during follow up. Our results are in agreement with those of recent larger RCTs that did not observe any modulation of CRP levels after CPAP therapy [36,38,39,40]. In contrast with these findings, there are studies which demonstrated a beneficial effect of CPAP on inflammatory markers [41,42,43]. Once again, CRP is only part of the complex inflammatory process that characterizes OSA. If these biomarkers are more closely related to obesity than to OSA, CPAP will only play a minor role in improving their levels.

### 4.3. Effect on Blood Pressure

There is evidence that OSA is a risk factor for the development of systemic hypertension [5]. Current guidelines point out that even a minor reduction in blood pressure can have clinical significance by reducing the associated cardiovascular mortality [44]. In our study we found that diastolic blood pressure levels were significantly improved by CPAP therapy, although with no significant effect on systolic blood pressure. In accordance with our results, Garcia Martinez et al. [45] showed that after 12 weeks of using CPAP therapy in OSA patients with resistant hypertension, diastolic blood pressure decreased but only a moderate reduction was observed in 24-h SBP in an intention-to-treat analysis. Similar results were obtained by another group [46] and in the SAVE (Sleep Apnea Cardiovascular Endpoint) trial [6]. In contradiction to our results, a small RCT showed an impressive 10 mm HG 24 h SBP reduction in the CPAP group [47]. The results obtained in our study, could be explained by the fact that DBP is more associated with OSA than SBP, which makes it more susceptible to CPAP therapy. Moreover, others [48] demonstrated that high AHI was independently associated with elevated DBP, finding no association between SBP and AHI. Generally, high SBP is assigned to stiff, noncompliant arteries, whereas elevated DBP is attributed to the activation of the sympathetic autonomic nervous system [49,50]. This pathogenetic hypothesis is consistent with our results and in agreement with the findings of other groups [51].

### 4.4. Effect on Ventricular Function

Based on previously described association of OSA with right ventricular dysfunction [52], one of our goals was to evaluate if treatment with CPAP could improve echocardiographic parameters, this being another potential step in reducing the cardiovascular risk in OSA patients.

The exact mechanism by which sleep apnea affects heart function is not fully elucidated, though it is thought to be largely due to pulmonary hypertension (PHT) [53].

We found that CPAP therapy improved RV systolic function by significantly increasing TAPSE, a well-established marker of RV function [54]. Although the trans-tricuspid gradient did not show improvement at follow up, it was slightly reduced when compared to baseline values in the CPAP group. There are several studies which confirm these findings [55,56,57].Karamanzanis et colleagues [58] showed partially similar results, the differences between the findings consisting of the effect of CPAP on RV diameter and LVEF, parameters which did not improve through our follow up period. In an observational study [59], the group showed that 6 months of CPAP therapy improved pulmonary hypertension in OSA patients, these results being in accordance with other studies [60]. Furthermore, increased pulmonary vascular reactivity to hypoxia was reversed by CPAP in patients with OSA and concomitant pulmonary hypertension [61]. On the other hand, many of these studies lacked invasive hemodynamic assessment, so patients included did not necessarily have significant pulmonary hypertension at study entry [60].

More recently, in accordance with our study, others showed [62,63] improvement in RV function as assessed by TAPSE over 12 weeks of CPAP usage.

It has also been argued [64] that conventional echocardiographic parameters, such as TAPSE, RV and MPI (myocardial performance index), are not significantly impaired in OSA patients, meaning that they may not be sufficiently sensitive in detecting subclinical RV dysfunction. In this case, CPAP treatment would have no effect on improving heart function in this category of patients. Similar conclusions were seen in [65], which reports no change in diastolic function and filling pressures by CPAP in a group of patients with OSA and heart failure.

Concerning the effect of CPAP on LVEF, things remain difficult to predict due to the fact that LV systolic functions depend on LV preload, afterload and compliance [66]. Previous publications have yielded inconclusive results [67]. In our study, no significant effect of CPAP was observed on LVEF after adjusting for confounding factors, while others describe an improvement of LVEF [68] in patients with associated sleep-disordered breathing (83% OSA patients) and chronic heart failure following CPAP therapy. A possible explanation for our negative finding regarding the effect of CPAP on LVEF could be the fact that participants with clinical significant LV dysfunction were excluded.

### 4.5. Lifestyle and CPAP Effect on BMI

It is well-known that OSA is most common in people who are overweight or obese. While obesity has long been thought to be a risk factor for OSA, there is evidence suggesting the association is reciprocal. This is because sleep deprivation is associated with decreased leptin (appetite-suppressing hormone) [69] and increased ghrelin (appetite-stimulating hormone), which may lead to cravings for high-calorie food [70]. The relationship between OSA and obesity is further supported by the results of Nedeltcheva et al. [71], who showed that people with OSA gained significantly more weight compared to BMI-matched patients without OSA in the year leading up to their OSA diagnosis. In our study, the majority of patients included were obese, with no significant differences in mean BMI, at baseline between the two groups, which confirms once again that obesity and OSA are strongly associated. Furthermore, at follow-up we observed a significantly reduction in BMI in the CPAP group, results being in concordance with those of Harsch et al. [72] suggesting that OSA patients who effectively manage their sleep apnea may find it easier to lose weight.

Lifestyle changes are the cornerstones of OSA therapy. Evidence from epidemiological research shows that people who are physically active and follow calorie-restricted diet have a reduced risk of OSA compared to individuals who are less active and with lower dietary quality [73,74]. Although exercise is a method of reducing body weight, there are meta-analyses on the effects of aerobic exercise on OSA, which show improvement in sleep quality, daytime sleepiness and AHI [75]. Although diets and exercise have major implications for cardiovascular morbidity associated with OSA, concomitant use of CPAP may end in more beneficial cardioprotective outcomes. Future studies should focus not only on one mechanism responsible for improvements in OSA following exercise training, but on several possible mechanisms.

### 4.6. Limitations

Some limitations of our study include the initial exclusion of patients with severely reduced EF, history of ischemic disease, cardiopulmonary decompensated disease and uncontrolled hypertension. Thus, our results may only be applied to populations that are similar to those in our study. Second, due to lack of statistical power, more patients need to be included to reach a certain conclusion regarding inflammatory markers, triglycerides levels, and LV EF. Third, blood pressure values were measured during clinic visits, an approach that can be influenced by observer bias and white coat effect, even if the measurements were taken following international guidelines. Fourth, we did not make special dietary recommendations or monitor the participants’ diets during the follow-up. This could be a limitation since changes in diet can have effects on lipid profile. Therefore, further studies with a larger number of participants and longer follow-up periods may be needed to investigate other parameters.

## 5. Conclusions

We confirmed that CPAP therapy independently decreases TC levels, DBP and ameliorates RV function by improving echocardiographic parameters such as TAPSE and, to a lesser extent, the transtricuspid gradient. Even though no effect was observed on TG and CRP levels, SBP and LVEF, in the light of the positive results obtained we can safely appreciate that CPAP treatment of OSA syndrome may play a key role both in the control and prevention of cardiovascular risk factors and their consequences on echocardiographic parameters.

In brief, as far as we know, this is the first prospective study in Romania evaluating a one-year impact of OSA therapy on specific cardiovascular parameters. The study provides support for better characterization and understanding of OSA implications in Romanian patients, with better risk stratification and importance/utility of CPAP therapy, thus having the potential to improve patient stratification and management.

## Figures and Tables

**Table 1:** Baseline Characteristics of all Randomized Patients.

Variable	Non-CPAP Group (n = 57, 34.9%)	CPAP Group (n = 106, 65%)	*p* Value
Age years	59 (52.6; 65.2)	61 (53.7; 67)	0.76
Sex female, n, %	26 (45.6%)	38 (35.8%)	0.22
BMI kg-m^2^	38.1 (34.4; 40.7)	37.5 (33.08; 41.9)	0.44
Additional cardiovascular risk factors:			
-DM type 2, n, %	18 (31.6%)	39 (36.8%)	0.5
-Arterial hypertension, n, %	39 (68.4%)	83 (78.3%)	0.16
-Smoking, n, %	37 (64.9%)	57 (53.8%)	0.17
-Alcohol, n, %	6 (10.5%)	10 (9.4%)	0.8
Medication:			
Lipid lowering drugs, n, %	22 (38.6%)	37 (34.9%)	0.08
Anti-hypertensive agents, n, %	39 (68.4%)	83 (78.3%)	0.16
SBP average, mmhg	145 (135; 157.5)	136 (130; 145)	<0.001
DBP average, mmhg	89 (80; 90)	82 (75; 89)	0.01
Serum levels lipids/CRP:			
Cholesterol levels mg/dl	199 (177; 218)	205 (166; 236)	0.4
Triglyceride levels mg/dl	160 (119; 225)	195 (118.5; 233.7)	0.9
CRP, mg/L	5.6 (3.4; 14)	5.8 (3.4; 11)	0.26
Results of the sleep study:			
AHI h^−1^	44.3 (22.8; 82.1)	45 (32.2; 65.9)	0.93
ODI h^−1^	43.2 (27; 80)	44.9 (27.3; 62)	0.55
Max O2 desaturation, %	72 (67; 78)	68.5 (60.5; 79.2)	0.61
Apnea average duration, s	73.5 (55.5; 98)	75 (49; 95)	0.4
Hypopnea average duration, s	84 (62.5; 101.5)	79.5 (56; 98)	0.14
Apneas, n	72 (56; 101.5)	71 (45; 103)	0.059
Hypopneas, n	98 (73.5; 120)	94.5 (71; 111.7)	0.7
Echocardiographic parameters			
RV, mm	35 (31.5; 39)	37 (35; 40)	0.16
TAPSE, mm	20 (17.5; 21)	19 (18; 21)	0.31
RA-RV Gradient, mmhg	30 (20; 40)	35 (24; 36.5)	0.9
LVEF, %	50 (50; 51.5)	50 (45; 55)	0.24

Data are presented as median (25–75 percentiles) and n (%); CPAP: continuous positive airway pressure, BMI: body mass index, AHI: apnoea-hypopnoea index, ODI, overnight desaturation index, SBP: systolic blood pressure, DBP: diastolic blood pressure, CRP: C-reactive protein, DM: diabetes mellitus, RV: right ventricle, TAPSE: tricuspid annular plane systolic excursion, RA: right atrium, LVEF: left ventricle ejection fraction.

**Table 2:** Effect of Continuous Positive Airway Pressure Treatment on the evolution of studied parameters.

	Without CPAP		With CPAP		
Variable	Baseline	Follow Up	Baseline	Follow Up	*p* Value
AHI h^−1^	44.3 (22.8; 82.1)	32 (16.5; 38.1)	44.3 (22.8; 82.1)	5.1 (2.1; 9.1)	<0.001
ODI h^−1^	43.2 (27; 80)	31.2 (16.8; 42.7)	44.9 (27.3; 62)	4.7 (2; 8.6)	<0.001
Apnea average duration, s	73.5 (55.5; 98)	31 (12; 24)	75 (49; 95)	12 (8; 32)	0.01
Hypopnea average duration, s	84 (62.5; 101.5)	52 (25; 68)	79.5(56; 98)	25 (15.7; 49.2)	0.03
Apneas, n	72 (56; 101.5)	23 (5; 63.5)	71 (45; 103)	8.5 (2; 23)	<0.001
Hypopneas, n	98 (73.5; 120)	56 (13; 79)	94.5 (71; 111.7)	15 (12; 36)	0.001
Max O2 desaturation, %	72 (67; 78)	84 (75.5; 87)	68.5 (60.5; 79.2)	90 (83; 94)	0.03
BMI, kg-m^2^	38.1 (34.4; 40.7)	37.2 (32.6; 39.7)	37.5 (33.08; 41.9)	35.2 (31.09; 40.3)	0.001
Cholesterol levels, mg/dL	199 (177;218)	190 (180; 222)	205 (166; 236)	175.5 (145; 210)	0.001
Triglyceride levels, mg/dL	160 (119; 225)	159 (119; 193)	195 (118.5; 233.7)	150 (104.5; 186.75)	0.2
CRP, mg/L	5.6 (3.4; 14)	7 (4.3; 8.7)	5.8 (3.4; 11)	3.1 (2.1; 9.4)	0.1
SBP average, mmHg	145 (135; 157.5)	135 (129; 145)	136 (130; 145)	125 (120; 130)	0.5
DBP average, mmHg	89 (80; 90)	80 (75; 90)	82 (75; 89)	75 (70; 80)	0.001
RV, mm	35 (31.5; 39)	36 (32; 39)	37 (35; 40)	37 (35; 40)	0.4
TAPSE, mm	20 (17.5; 21)	19 (18; 21)	19 (18; 21)	20 (19; 21)	0.03
RA-RV Gradient, mmhg	30 (20; 40)	28 (20; 39)	35 (24; 36.5)	34 (25; 38.5)	0.01
LVEF, %	50 (50; 51.5)	50 (50; 55)	50 (45; 55)	50 (45; 55)	0.8

Data are presented as median (25–75 percentiles), CPAP: continuous positive airway pressure, BMI: body mass index, AHI: apnea-hypopnea index, ODI, overnight desaturation index, SBP: systolic blood pressure, DBP: diastolic blood pressure, CRP: C-reactive protein, RV: right ventricle, TAPSE: Tricuspid annular plane systolic excursion, RA: right atrium, LVEF: left ventricle ejection fraction.
